# Multiple Deprivation, Severity and Latent Sub-Groups: Advantages of Factor Mixture Modelling for Analysing Material Deprivation

**DOI:** 10.1007/s11205-016-1272-y

**Published:** 2016-02-22

**Authors:** Hector E. Najera Catalan

**Affiliations:** School for Policy Studies, University of Bristol, Bristol, UK

**Keywords:** Deprivation, Poverty, Severity, Latent class analysis, Factor mixture model

## Abstract

Material deprivation is represented in different forms and manifestations. Two individuals with the same deprivation score (i.e. number of deprivations), for instance, are likely to be unable to afford or access entirely or partially different sets of goods and services, while one individual may fail to purchase clothes and consumer durables and another one may lack access to healthcare and be deprived of adequate housing . As such, the number of possible patterns or combinations of multiple deprivation become increasingly complex for a higher number of indicators. Given this difficulty, there is interest in poverty research in understanding multiple deprivation, as this analysis might lead to the identification of meaningful population sub-groups that could be the subjects of specific policies. This article applies a factor mixture model (FMM) to a real dataset and discusses its conceptual and empirical advantages and disadvantages with respect to other methods that have been used in poverty research . The exercise suggests that FMM is based on more sensible assumptions (i.e. deprivation covary within each class), provides valuable information with which to understand multiple deprivation and is useful to understand severity of deprivation and the additive properties of deprivation indicators.

## Introduction

Poverty can be defined as a lack of resources over time, and deprivation as its consequence (Townsend 1979; Gordon 2006). In poverty research a number of studies have aimed at estimating, on a valid and reliable basis, the prevalence of deprivation or poverty in a given society (Townsend 1987; Halleröd 1995; Atkinson 2003; Whelan and Maitre 2006; Gordon 2006; Alkire and Foster 2011).

Much less attention has received the topic of severity of deprivation and the analysis of multiple deprivation. A number of poverty measures draw on Townsend’s (1979) deprivation score (also known as the counting approach in the literature (Atkinson 2003)), which is a parsimonious way of classifying a population according to the number of deprivations and helps to analyse the distribution of its severity among people. The main assumption of this index is that deprivations are additive and a higher number of deprivations is associated with higher severity. Therefore, it is also a useful measure of severity.

Townsend (1987) pointed out that the deprivation score is not useful in analysing multiple deprivation and more refined analyses are required to be understood fully. For example, people or households may be unable to afford or access different goods and services, and the same score is likely to hide a wide range of situations-two individuals with the same deprivation score do not necessarily lack the same things. The deprivation score therefore is inadequate for capturing the complexity of multiple deprivation, as it involves several sequences or combinations of deprivations. This complexity can be illustrated crudely as follows: if a deprivation index is derived from ten dichotomous indicators, there are up to $2^{10}=1,024$ possible sequences or combinations, each of which follows a binomial series. There are additional questions about how the degree of severity varies across different combinations of deprivations, for example whether a person lacking water and sanitation is deprived as equally as one lacking regular access to food and sanitation. In this regard, Townsend (1987) pointed out that it is vital to understand the patterns of deprivation in order to answer questions such as: *What deprivations characterise the severely deprived and the mildly deprived? How can such patterns be related to existent policies? Why are some combinations of deprivations more likely to affect certain populations? How does multiple deprivation relate to the severity of material deprivation?*

The heterogeneity of multiple deprivation has opened up questions in contemporary poverty research about how to simplify such patterns and find meaningful and well-interpretable—from a policy perspective—population sub-groups (see for example Moisio 2004; Whelan and Maitre 2005; Machado et al. 2009). The literature on multiple deprivation has relied mostly on Latent Class Analysis (LCA) to infer from the data the number and type of sub-groups (see Sect. 3). Although this approach has attractive properties (model selection), recent development in statistical literature permit to overcome some limitations of LCA, such as the assumption of conditional independence, assuming that deprivation is discrete and lacks the possibility of estimating the severity of deprivation for each case in the sample (see Sect. 4) (Magdison and Vermunt 2004).

Factor mixture models (FMMs) provide a hybrid framework that combine a common factor model with LCA, and offer a more powerful framework to analyse multiple deprivation (Lubke and Muthén 2005; Muthén 2007; Reboussin et al. 2008). However, its limitations and advantages have not been yet discussed in the context of poverty research. The purpose of this article, therefore, is to contribute to current studies on the analysis of population heterogeneity and multiple deprivation (Lubke and Muthén 2005; Muthén and Shedden 1999). This model allows deprivations to covary within each class (conditional dependence), tests whether derivation is discrete or continuous and permits to analyse the relationship between severity and difference sequences (see the next sections for a discussion). Moreover, it uses data from the Mexican Household’s Income Expenditure Survey 2012 (or ‘ENIGH’—its acronym in Spanish) as a motivating example.

The article is organised as follows. Section 2 reviews the literature on the empirical analysis of multiple deprivation, and Sect. 3 discusses the virtues and advantages of current statistical approaches in poverty research. Section 4 describes and presents the formulation of factor mixture models (FMMs), while the following Sect. 5 provides an application of an FMM using the Mexican survey. Finally, Sect. 6 discusses the findings and concludes the article.

## Literature on the Analysis of Latent Sub-Groups and Multiple Deprivation

There has been increasing interest in the analysis of multiple deprivation and the identification of latent sub-groups in the European literature, though virtually no studies have been carried out which consider developing countries, where material deprivation is more extreme and deprivation is rife in many areas (see for child poverty Gordon et al. 2003).

In the European context, Whelan and Maitre (2005), using data from 13 European countries and a latent class model (LCA), explored the existence of a distinguishable and latent population sub-group vulnerable to economic exclusion. The authors use three measures of exclusion, namely material deprivation, income poverty and economic strain, and also analyses the set of predictors for each class. This is done as a second step in fitting a regression model, by using the sub-groups obtained from the LCA model. According to the findings, socio-economic factors such as being a lone parent and divorced are associated positively with the likelihood of being economically excluded in Europe. Similarly, proxies of class membership were utilised as predictors of economic exclusion, the results suggesting that manual workers are considerably more likely to be part of the economically excluded class. Moreover, there is a negative association between the upper classes and the chances of being part of the economically excluded latent group. A similar approach was undertaken by Whelan and Maître (2007) in an analysis of Ireland, using EU Statistics on Income and Living Conditions survey. Using a latent class model helped in identifying a group of economically vulnerable people and another sub-population vulnerable to maximal deprivation.

Halleröd and Larsson (2008) explored the relationship between poverty and welfare problems, by looking at the connection between poverty and phenomena such as material deprivation, economic precariousness, unemployment, psychological strain and health problems in Sweden in the late 1990s. Drawing on theories of cumulative disadvantage (Paugam 1996; Gallie et al. 2003), they investigate situations where people suffer from different problems simultaneously. As in other studies, and given the large number of combinations or sequences of deprivations, from the statistical point of view, their interest is in elucidating whether latent sub-groups can be inferred from the data. They use Statistics Sweden’s Annual Survey of Living Conditions from 1998, and similarly to Whelan and Maitre (2005) and Whelan and Maître (2007), they analyse population heterogeneity using latent class analysis with a subset of indicators (eight indicators for the final model). According to their findings there were three latent sub-groups with distinguishable welfare problems: one with virtually no welfare problems, a second group likely to suffer from health and unemployment and a third group with the most welfare problems. As a second step, they used a multinomial model to look at the predictors for each class.

Dewilde (2004), using data from Belgium and the UK, carried out a confirmatory two-latent class analysis to identify whether a set of indicators of deprivation resulted in a set of domains measuring the same construct. A similar approach was utilised by Dewilde (2008) to analyse multidimensional poverty in Europe and its relationship with institutional and individual determinants. Similarly, although concerned more with classifying indicators rather than the population, Moisio (2004) utilised latent class to analyse the validity and reliability of a set of deprivation indicators for measuring poverty on a multidimensional basis in Finland, the Netherlands and the UK.

More recent studies have incorporated more complex methods into identifying sub-groups with different profiles of multiple deprivation. For example, Machado et al. (2009) employed a Bayesian latent class model to analyse multiple deprivation from a dynamic perspective among Portuguese households. On the basis of indicators stretching across four dimensions (housing, durable goods, economic strain and social relationships), they compared changes in multiple deprivation for four latent classes between 1995 and 2001. The classes they found varied in both their severity of deprivation and the items of which were more likely to be deprived. Pirani (2013) utilised a random effects latent class model to analyse social exclusion in Europe and found a range of exclusion structures for different regions. This analysis was undertaken using data from the Eurobarometer (EB) 2001, which enabled the authors to include 77 regions for 15 European countries. According to their results, six classes and four regional clusters were identified, with the latter being scattered among countries.

Others studies have attempted to relax the assumption of local independence. For example, Whelan et al. (2010) recently acknowledged that the conditional independence assumption made in an LCA might be too simple, and they used self-organizing maps (SOMs) to analyse multiple deprivation. They found the existence of 13 clusters of multiple deprivation, which later they grouped into eight distinguishable clusters (Kohonen 1982). Each of the clusters is characterised by the main deprivation, for example, cluster of multiple deprivation least pronounced in health. Using as a reference the work of (Whelan and Maître 2007), they compared their results with those obtained from an LCA and found that for their particular dataset the SOM analysis offers more discriminatory power, albeit there are similarities between both approaches.

## Methods for the Study of Multiple Deprivation

The analysis of multiple deprivation has a statistical counterpart in the study of *population heterogeneity*. In social sciences there are different features or characteristics of the population that are observable or unobservable. For example, gender, age or other socio-demographic characteristics are explicit markers of observed population heterogeneity. In other cases, from a conceptual perspective, a population is assumed to be heterogeneous due to an unobserved condition. Social class is a common example in which a series of attributes of the population are utilized to classify each individual in a specific class. In poverty research, a population is divided into groups such as *deprived, not deprived, poor and not poor*. Unlike in the first case, these concepts underlay a resulting classification of the population. Furthermore, such groupings, inasmuch as there are unobserved a priori, require to be identified on the basis of existent data.

Several statistical methods have been proposed to analyse population heterogeneity (Meehl 1992; Lubke and Muthén 2005). When the sub-populations are known beforehand (i.e. observable heterogeneity) Generalized Linear Models (GLMs) are appropriate to investigate how a given response varies according to group membership. Discriminant analysis, Multivariate Analysis of Variance (MANOVA) or multi-group Confirmatory Factor Analysis (CFA) can be also be used as alternatives (Jöreskog 1971; Nelder and Wedderburn 1972; Wooldridge 2005). This analysis often aim to assess how a certain measure varies across population (e.g. Are women more likely to be poor than men?)

Unobserved heterogeneity posses a rather different challenge. A theory could suggest that there are two groups: not poor and poor, for example. But the existence of these two needs to be validated from an empirical perspective. In other cases, when there is no theory, it is necessary to explore the possible existence of groups. The literature review showed that often this is the case when studying multiple deprivation. This raises the question about what kind of data-reduction technique is adequate to analyse multiple deprivation.

When population membership is unknown, cluster analysis, Latent Class Analysis (LCA), latent profile analysis or self-organising maps (SOMs) are adequate (Lazardfeld and Henry 1968; Kohonen 1982; Everitt et al. 2011), while hybrid models, such as factor mixture models (FMMs), have been recently proposed to overcome some of the shortcomings of the methods mentioned above (see discussion below)(Lubke and Muthén 2005; Muthén 2007).

All current methods have advantages and limitations, and most that is known about them is based on evidence derived from simulated datasets. The literature has found that cluster analyses tend to perform better in a two-staged design, where results from hierarchical clustering (Ward method for example) are employed to sow the seeds for a k-means analysis (Zimmerman et al. 1982; Dubes 1987). However, there is not an unequivocal criterion that can be used to choose the best solution (Milligan and Cooper 1985; Atlas and Overall 1994). The scope of this limitation nonetheless depends on the interest of the research, as in an exploratory setting cluster analysis is likely to provide useful information. When compared with other methods, particularly with model-based approaches, Magidson and Vermunt (2002) have shown that LCAs outperform cluster analyses. In particular when the conditions of the experiment should favour a k-means analysis.

In recent years, the LCA has been the preferred method for either exploring or confirming population heterogeneity in the context of multiple deprivation (Lazardfeld and Henry 1968)(see Sect. 2). Since this approach is model-based, one of its most attractive features is that by comparing the fit of the models it is possible to assess which option (i.e. number of groups) best fits the data (Lubke and Tueller 2010; Lin and Dayton 1997). Local independence is one of the main assumptions of LCA (Magdison and Vermunt 2004). A consequence is that, once the number of latent sub-groups has been identified, within-class covariances of the indicators are zero (Lazardfeld and Henry 1968; Clogg and Goodman 1984). In other words, people is assigned to a series of location points, and at each point it is presumed that all have the same degree of severity of deprivation. That means, for example, that the relationship between poor sanitation and malnutrition is entirely explained, plus the error, by group membership (i.e. being severely deprived) and not for other kind factor.

The literature has pointed out that a statistical limitation of LCA is that model fit is conditional to achieving local independence. An unfortunate consequence is that model fit improves as more classes are added to the model. For example, when local independence does not hold for *k* number of classes (i.e. group membership does not account for all the relationship between indicators), LCA will inflate the number of groups as adding *k+1* classes will (artificially) introduce more locally independent groups. When local independence does not hold, a standard LCA is likely to fail to find the true number and configuration of latent sub-groups (Harper 1972; Uebersax 1999). Hence, there is the risk of finding meaningless classes, i.e. dividing a class that was meaningful into two non-interpretable groups that affect the overall fit.

Local independence is unlikely to hold in practice (Harper 1972; Uebersax 1999; Magdison and Vermunt 2004), as group membership is often insufficient to account for the relationship between the indicators. In the context of poverty research, one prediction that can be derived from Townsend’s (1979) theory is that deprivations are correlated among different population groups (observed or unobserved) (Guio et al. 2012). For example, in a class where people are severely deprived, it seems unreasonable to assume that deprivations do not covary in some way. Furthermore, local independence also implies that the observed responses are independent of covariates (non-differential measurement), which is an important limitation, as LCAs in poverty analysis are often used for prediction (e.g. which socio-economic characteristics predict the fully deprived group).

Psychometric literature has raised other kinds of concerns about LCA models. In particular, LCA assumes that a phenomenon is discrete rather than continuous (Muthén 2007). This means that a population can be classified into three groups, for example, severely, moderately and mildly deprived and that within each group the degree of severity is exactly the same. Drawing on the work of Townsend (1979) and Gordon et al. (2003) conceptualise deprivation as a continuum, ranging from mild to severe deprivation. On the basis of available data a threshold is chosen to identify the deprivation of a given item. However, this does not mean that it is a discrete process, because in most studies that have investigated population heterogeneity, deprivation is treated as a discrete process, which means that the severity of deprivation within a class is exactly the same for all members of that class, regardless of whether they suffer from different deprivations. Moreover, it is not possible to know how severity varies between classes.

Concerns about local independence have been raised in several fields, and different solutions have been proposed (Reboussin et al. 2008; Uebersax 1999). However, these methods have four disadvantages. First, they require the joint estimation of three separate models for each solution of the common LCA. Second, it is necessary to use covariates (predictors) to adjust the estimates. Although this is not a limitation in itself, the analysis of the predictors of each class is normally seen as a second step (e.g. what factor is associated with being severely deprived?) Third, severity of deprivation is assumed to be a discrete phenomenon. These approaches do not offer the possibility of computing severity of deprivation for different combinations of items, which is necessary to understand better the relationship between multiple deprivation and severity—it is not possible to know whether the sum of A and B lead to a higher severity in comparison to the sum of item B and C.

Factor mixture models (FFMs) relax the assumption of local independence and enable within-class correlation of the observed items, and because FMMs are based on a common factor model with classes, they conceptualise deprivation as a continuum (Muthén and Shedden 1999; Muthén 2007; Lubke and Muthén 2005). One of the main advantages of FMM’s is that they provide valuable information about severity of deprivation. For example, it is possible to know the level of severity for combinations of items. It is possible to compute the level of deprivation for a household lacking water and regular access to food. Then this value can be contrasted against households with a different pattern (e.g. health care and sanitation) or with a different deprivation score. Comparisons of the mean or of the variance of severity are possible with this approach; moreover, simulations have shown that under many conditions FFMs tend to outperform LCAs, as they lead to selecting the correct number of groups (Lubke and Tueller 2010; Lubke and Neale 2008, 2006).

### Factor Mixture Model Formulation

The model formulation is very similar to a common factor model (Thurstone 1947), in which a latent variable (deprivation) is measured by a series of manifest variables (observed deprivations). Following the formulation of Lubke and Muthén (2005) the common factor model is expressed as:

1 $$y_j=\nu + \varLambda _y\eta _i + \varGamma _yx_i + \epsilon _i$$

2 $$\eta _i=\varGamma _{\eta }x_i + \zeta _i$$

where *Y* is a vector containing the items through which deprivation is measured, $\nu$ is the intercept and $\varLambda _y$ are factor loadings. Equation (2) represents a regression of the factor scores $\eta _i$ on the covariates $x_i$, where $\zeta _i$ is a residual not explained by the covariates and $\varGamma _{\eta }$ are regression weights.

Equations (1) and (2) denote an FMM with one class, which can be extended for *k* classes as follows:

3 $$y_{jk}=\nu _k + \varLambda _{yk}\eta _{ik} + \varGamma _{yk}x_{i} + \epsilon _{ik}$$

4 $$\eta _{ik}=Ac_i + \varGamma _{{\eta }{k}}x_i + \zeta _{ik}$$

The main difference between Eqs. 1–4, is that they incorporate a Latent Class Model (LCA) into a common factor model. This is akin to the idea of having predictors such as gender or age in a factor model. In this case, the latent classes have the same function as the observed variables. The subscript *k* is included, in order to allow the parameters to vary across classes. It is important to note that the observable predictors do not vary across classes, because the model is estimated conditional on the covariates. This is a very useful feature as in one model is possible to analyse the predictors of each class, which, as explained above, it is often a research goal in the analysis of multiple deprivation.

FMMs with several classes, where the parameters do not vary across classes, are just a common LCA. This feature is important because it offers the possibility of comparing the fit between different models (Nylund et al. 2007; Lubke and Muthn 2007). Therefore, it is possible to assess whether is using a FMMs is suitable in comparison o a more parsimonious model.

Factor mixture models nevertheless several disadvantages that can be relevant depending on the characteristics of the data. First, the structure of the factor (i.e. the latent configuration of deprivation) needs to be specified following a confirmatory setting (Confirmatory Factor Model, CFA). Although an exploratory FMM could be fitted, this would lead to several solutions which largely depend on the different factor structures. This limitation is related to a second drawback whereby if a multidimensional measure or factor needs to be specified, this would involve a major computational challenge, as FMMs require maximum likelihood, and each first-order factor (i.e. deprivation dimension) would require one dimension of numerical integration.

A third issue involves the statistical principle of *measurement invariance* (MI) (Meredith and Teresi 2006) whereby the same construct is measured equivalently across classes. When MI is violated it is invalid to compare the scores or an index between two sub-populations, for example if an indicator on food deprivation is found to be non-invariant between two countries. Furthermore, comparisons on the basis of such an indicator are not very informative about which country experiences more or less food deprivation, as this concept is measured differently in each unit.

Because FMMs are very flexible, there is the risk of violating MI (Lubke and Muthn 2007; Lubke and Muthén 2005), due to the fact that the parameters of the model can vary across classes. Therefore, factor loadings (slopes) and thresholds (intercepts) might be different within each class, which would affect the comparisons between classes. On the other hand, FMMs have the advantage of permitting comparisons of the factor mean, which is very useful in the case of the analysis of deprivation, as it is possible to know which group is the more severely deprived. Drawing upon (Meredith and Teresi 2006), Lubke and Muthén (2005) propose a list of cases in which MI might be an issue in the context of FMM.

Weak factorial invariances hold when the slopes (i.e. factor loadings) are identical across sub-groups. When this does not hold, though, an increase in the severity of deprivation is not related to the same score observed across sub-populations, which would imply the absence of a factor in the deprivation index.

A more restrictive form of MI is strong factorial invariance. It holds when both the loadings and the intercepts are equal cross sub-populations. When the intercepts are not identical, this means that one sub-group is consistently more deprived than others in a given item. Finally, strict factorial invariance implies that there are equal loadings, intercepts and residual covariances. In practice, strong factorial invariance is considered as sufficient for the comparison of sub-populations. This is due to the fact that the equality of loadings and intercepts ensures that observed differences in the mean between two sub-populations are due to differences in the factor.

### Data

In order to illustrate the application of an FMM and how its results compare with those for an LCA, this article uses data from the Household Income and Expenditure Survey (Mexico 2012) (ENIGH, by its acronym in Spanish) (INEGI 2012). The ENIGH is a complex survey representing national, urban and rural areas for 32 Mexican states. The sample size is 212,674 cases.

For the purpose of the article, it was decided to draw on a measurement model. Therefore the FMM draws on a CFA model. The official Mexican Multidimensional Poverty Measure (MPM) combines, using the intersection method, direct (deprivations) and indirect (income) measures to estimate poverty (CONEVAL 2011, 2009). For this article, only the direct (dichotomous) indicators are used (as the focus is on multiple deprivation). The use of binary variables is on purpose as many deprivation measures are based on dichotomous variables. However, in some case they are produced after transforming nominal or ordinal variables, as it is the case with the MPM. The thresholds of some indicators differ from those utilised to construct the official measure. The reason for such changes is that some of them measure very severe aspects of deprivation and affect the validity and reliability of the measure (the Item Characteristic and Total Information Curves of the unadjusted measure can be obtained from the author). Following the type of coding in poverty research, lacking an item is equal to 1 and having the item equal to zero. So that the deprivation score reflects the degree of severity.

The dimensional structure of the MPM is akin to a second-order factor. That is, a higher order factor (poverty), measured by two domains (or dimensions): deprivation (also known as the social rights or standard of living dimensions within the Mexican context) and welfare (income poverty) (CONEVAL 2011). Deprivation (direct measurement) is related to a set of basic socio-economic rights set out by Mexican legislation: compulsory education, access to health and social security (minimum social protection floor), access to essential public services, food deprivation and adequate housing deprivation.

- Food deprivation: People suffering 3 or more hunger episodes. Mild food insecurity according to (CONEVAL 2011)
- Access to a minimum social protection floor: People lacking health care or social security.
- Inadequate flooring: Lacking a floor made of cement, tiled or laminated.
- Inadequate roofing: Lacking a roof made of cement, slab, roof with beams or tile.
- Inadequate walls: Lacking walls made of cement, brick or block.
- Overcrowding: More than 2.5 per room.
- Access to water: Lacking water everyday.
- Sanitation: Lacking and independent toilet connected to water.
- Lacking education: People without secondary education or who are not in education (people aged 16 or less). (See (CONEVAL 2011) for details about the normative ages for different cohorts)

It is important to distinguish the difference between focusing on multiple deprivation and looking at multiple deprivation an income. Whereas in the first case the focus is on simplifying the number of patterns of material deprivation and relate them with severity, in the second case the focus would be on assessing the intersection method (income and deprivation) as well as the relationship between different levels of income and material deprivation. From a methodological perspective, this poses a serious computational challenge as a two dimensional (deprivation and income) higher-order mixed-model containing binary and continuous variables is required. There is, as will be shown below, a descriptive approach to analyse the association of multiple deprivation and severity with income.

### Statistical Models: Factor Mixture Model

Drawing on the strategy adopted by Lubke and Muthén (2005), in order to compare the results of different models (latent class model vs factor mixture model), less restrictive models (i.e. free parameters within a class) with an increasing number of latent classes were estimated. The models are as follows.

*Model 1* is a baseline model of the latent construct (i.e. overall deprivation), known as a two-parameter Item Response Theory model (IRT). It has been used widely in psychometrics and is an extension of the Rasch model (Rasch 1966). Although there are conceptual and mathematical differences between the Rasch model and the two-parameter IRT model, the Rasch model is often seen as a one-parameter IRT model (see for an introduction Hambleton and Jodoin 2003), and whereas in the Rasch model the key parameter is difficulty (i.e. the location of the item on the continuum of deprivation), the two-parameter IRT model adds the parameter of discrimination. In the context of the analysis of deprivation, difficulty indicates the severity of deprivation for a given item, and discrimination refers to how well an item distinguishes between the deprived and the not deprived. Such modelling has been applied recently for European data (Szeles and Fusco 2013).

IRT model assume unidimensionality. This seems to be untenable given that the MPM is multidimensional: two-order factor measure (see above). However, as previously noted, the focus of the article is on the standard of living dimension (deprivation). In this regard, a two-parameter item response model was estimated as the baseline model. IRT models can be seen as a unidimensional CFA model for categorical indicators. As has been shown, violations of the assumption of unidimensionality are not problematic in the presence of a higher-order factor, as the relative bias of the parameters is rather small (Drasgow and Parsons 1983). For these data, for example, a hierarchical omega $\omega _h$ statistic is above 0.8, indicating the presence of a higher-order factor (Revelle and Zinbarg 2009).

Model 2 comprises a set of LCMs with an increasing number of classes. In this regard, the analysis assumes the structure of the factor as given and is exploratory with respect to the number of classes. Model 3 comprises a series of FMMs with an increasing number of free within-class parameters (mean, variances, thresholds and factor loadings).

Model selection is based on statistics of fit, such as Akaike’s information criteria (AIC) and Bayesian information criteria (BIC). Simulation studies suggest that it is possible to choose the correct model when using continuous indicators and to choose between a model with local independence (LCA) and FMMs (Lubke and Neale 2006). Although the same holds for most comparisons when using categorical indicators, there are some problems when an FMM has several free parameters, in particular when thresholds are free to vary across classes (Lubke and Neale 2008). Regarding this second case, model comparisons are affected by the number of estimated parameters; therefore, when several parameters are allowed to vary across classes, there is the risk of selecting an incorrect model. The findings Lubke and Neale (2008) suggest that a more powerful statistic is required, particularly in those cases where the thresholds are free across classes. In this latter case, the AIC showed the best performance among the other statistics.

## Results

Table 1 shows the prevalence rate of deprivation for each of the ten indicators. As can be appreciated, the rates fluctuate a lot from indicator to indicator. Whereas lacking access to a minimum social protection floor is the most common deprivation among the Mexican population, only 4 % of the total population lacks adequate flooring material.

The Mexican official measure includes five indicators associated with the housing dimension. These indicators are associated with the severest forms of material deprivation (i.e. low prevalence rates, Table 1). However, the IRT model suggest that they tend to measure severities and therefore contribute to the measurement of deprivation in Mexico. The potential effect of this imbalance upon the results of the analysis (i.e. type and number of latent groups) is discussed below.

**Table 1:** % of population deprived in the given item (Mexico 2012)

Item (Deprivation)	Indicator deprivation prevalence rate %
Minimum social protection floor	62
Access to water	47
Food deprivation	44
Sanitation	40
Roofing materials	25
Education	19
Walling materials	14
Fuel	13
Overcrowding	10
Flooring materials	4

*Source*: Estimates based on INEGI-CONEVAL, 2012

For these 10 indicators there are up to 1,024 sequences or patterns ($d^n$, where *d* is 2 when using dichotomous variables and *n* the number of items $2^{10}=1024$). Table 2 shows the distribution of the deprivation score. As can be appreciated, around one out of every three people in Mexico is deprived, and almost half experience two or more forms of deprivation. According to Table 1 around 60 % of the population are deprived of minimum social protection floor, and therefore the other indicators are very likely to overlap with this item. In other words, people lacking two or more items are likely to lack a minimum social protection floor and another item.

When a deprivation score is based on less than three indicators it is relatively easy to check the sequences by using a three-way cross-tabulation or by using a three-way Venn diagram. However, given the number of possible patterns, such a descriptive strategy is not feasible. An alternative approach would be to calculate all the possible intersections of the items and create a list with the highest percentages of the most important overlaps. One limitation of this strategy is that the number of sub-groups could be difficult to handle, as such groups would not offer a parsimonious representation of the population and there would not be a clear criterion with which to select the number of groups and its configuration. Therefore, the results of such an analysis would be hardly reproducible and disagreement would exists regarding the correct number of meaningful latent sub-groups.

**Table 2:** Deprivation score and cumulative proportion (Mexico 2012)

Deprivation score	Percentages	Cumulative percentages
0	14	14
1	21	35
2	17	52
3	13	65
4	10	75
5	8	83
6	6	89
7	5	94
8	3	97
9	2	99
10	1	100
Total	100	100

Ten indicators/items

*Source*: Estimates based on INEGI-CONEVAL, 2012

## Results of Statistical Models

**Fig. 1:**
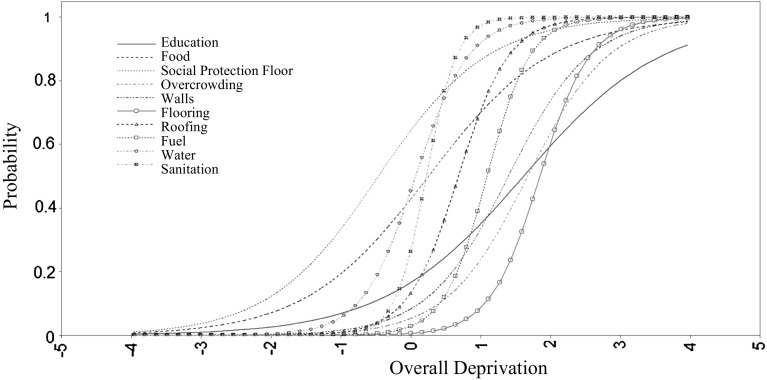
Model A: two-parameter IRT Model

The baseline model of the deprivation measure is a Two-parameter Item Response Theory (IRT) model. Figure 1 shows the Item Characteristic Curves (ICCs) of the IRT Model. Each curve is computed using the parameters discrimination and severity (difficulty) of the IRT model. Discrimination (i.e. the shape of the curve) indicates how well a given item distinguishes between the deprived and the not deprived. The steeper the curve, the better its discrimination. The severity is the position on the continuum—curves located on the right-hand side denote those items that characterise the severest form of material deprivation.

As can be appreciated, the curves tend to be closer to the mean of the factor (zero). However, they are not concentrated so much around the centre, indicating that the measure captures different degrees of deprivation severity. There are several items that would be helpful in distinguishing between the deprived and the not deprived. The indicator of sanitation seems to be the best candidate, while other indicators such as flooring and overcrowding seem to be more useful for distinguishing between the severely deprived and the not severely deprived.

Table 3 shows the goodness of fit statistics of the Latent Class Analysis (LCA) and the factor mixture models (FMM). Both the Akaike Information Criteria (AIC) and the Bayesian Information Criteria (BIC) lead to the same conclusions. As expected for the LCA the fit increases with the number of classes. A six-class model proved to be the best model among the five estimated models. As can be appreciated for the seven-class (7C) model, the best log-likelihood could not be replicated. Therefore the AIC and BIC were not reported as the estimates are unreliable .

The four-class FMM showed the best fit among all models. This indicates that allowing correlations between different deprivations within classes is a more reasonable approach in comparison to assuming local independence. One important result is that the thresholds are non-invariant across sub-groups. This is an indication the factor (overall derivation) does not explains differences across classes. There are unobserved factors (socio-economic characteristics or place) that explain why people in a given class is more or less likely to be deprived.

**Table 3:** Goodness of fit statistic

Model	AIC	BIC	Free parameters
Baseline model (IRT)	1855064.9	1855270.3	20
3C	1850764.8	1850991.7	32
4C	1842828.7	1843133.6	43
5C	1840151.2	1840534.0	54
6C	1838010.8	1838471.6	65
7C	NR	NR	NR
3C free mean	1855257.9	1855494.1	23
4C free mean	1854653.5	1854830.8	25
5C free mean	1854655.5	1854839.9	27
6C free mean	NR	NR	
3C free var and thresholds	1839136.9	1839441.7	45
**4C free var and thresholds**	**1837381**.**5**	**1837771**.**4**	55
5C free var and thresholds	NR	NR	NR
3C free mean and thresholds	1839136.9	1839578.3	43
3C free mean and slopes	1850782.8	1851203.8	41
4C free mean and thresholds	1838254.1	1838818.8	55
4C free mean and slopes	1842846.7	1843380.6	52
5C free mean and thresholds	1840179.2	1840661.3	68
3C free mean, var and thresholds	1839140.9	1839459.9	44
4C free mean, var and thresholds	NR	NR	NR

Factor mixture models

NR=The best log-likelihood was not replicated.

Bold values indicate the best model using Akaike Information Criteria (AIC). Bayesian Information Criteria (BIC)

Table 4 shows the value of the thresholds (intercepts) for each one of the four classes. They represent how likely each sub-population is to lack (negative sign) a particular deprivation. Following Muthén and Muthén (2012), values around −3.0 denote a very high likelihood of being deprived of a particular item. Conversely, values 3.0 indicate a very high probability of not being deprived of a particular item. Values around zero suggest a situation in which people is equally likely (50 %) of being deprived of a given item.

Class 1 seems to represent a group of multiple-severely deprived people (MSD). As can be appreciated this group is very likely to be deprived of Water (−2.8) and the Minimum Social Protection Floor (−2.7). They also are likely of lacking Sanitation, Fuel and, to some extent, Food (all negative values). They are equally likely of being deprived from adequate roofing and education (values near zero). Yet, they are very unlikely of lacking Walls and Flooring. It was mentioned that the imbalance of the number of indicators for measuring the housing dimension could affect the results. Given that the housing dimension is associated with the severest forms of deprivation (see item characteristic curves above), it is unlikely that dropping these indicators might modify the results as in all four classes the likelihood of being deprived of these items is low (they have positive thresholds).

The second class is also a multiple deprived sub-group, which comprises 34 % of the population. They have high chances of being deprived of sanitation and water and to some extent of the minimum social protection floor. The main difference between this group and the first class, is the lower likelihood of lacking fuel and education. On the basis of this table is rather difficult to compare the severity of deprivation between these two classes. This is explored in Table 5 (below).

The third class (Multiple Mildly Deprived) comprises people slightly likely of being deprived of minimum social protection floor and of food. They are slightly unlikely of being deprived of sanitation, water and education and have higher chances of living in a house made from inadequate materials. People in the fourth class are unlikely to be multiple deprived. This does not mean they are unlikely of not being deprived as they have almost equal chances of lacking the minimum social protection floor. There likelihood of not experiencing food deprivation or water deprivation is not as high as for the other items.

**Table 4:** Thresholds (likelihood of lacking the item) for the 4-class solution

Deprivation	Class 1 (MSD)	Class 2 (MMD)	Class 3 (MMiD)	Class 4 (NMD)
Food deprivation	−1.2	−0.5	−0.7	1.4
Education	0.34	1.0	1.4	2.4
M.S.P.F	−2.7	−1.5	−0.8	0.3
Overcrowding	1.6	2.0	2.1	5.6
Walls	2.7	2.0	3.0	3.6
Flooring	2.8	3.2	4.4	7.7
Roofing	0.4	.02	2.7	5.1
Fuel	−2.3	1.7	3.2	7.2
Water	−2.8	−2.4	1.2	1.5
Sanitation	−2.3	−2.4	1.2	3.0
Variance	Fixed	0.2	0.05	0.09
% total population	5 %	34 %	16 %	45 %

Values $\approx -3$ indicate high probability of lacking the item. $\approx +3$ not lacking

*MSD* multiple-severely deprived, *MMD* multiple-moderately deprived, *MMiD* multiple-mildly deprived, *NMD* not multiple deprived

If this results are sensible there should be a relationship between the type of class and the observed deprivation score (sums of deprivations). One way of analysing the relationship between class membership and severity of deprivation is by producing a simple cross-tabulation. Table 5 uses the deprivations score as measure of severity and compares whether the classes that are supposedly more severely deprived related with higher deprivation scores (as explained below with FMM’s is possible to compute a model-based deprivation score) .

Class 4, for example, comprises the not multiply deprived and it should be sensible to see that this class has low deprivation scores. As can be appreciated there is correspondence between the probability of not having deprivations and the deprivation score (zero). Class 3 represents the multiple-mildly deprived group. Around a third of the population with a deprivation score equal to two or three are likely to be classified as member of Class 3. People with more than four deprivations are very likely to be part of the group of the multiple-moderately deprived. Because only 5 % of the population was classified into Class 1, there is no clear relationship between the deprivation score and class membership. It is likely that what matter most is not the number of deprivation but the types of deprivations. As shown in Table 5, the main difference between Class 1 and 2 is that the former is more likely to comprise people lacking fuel, food and MSPF.

**Table 5:** Probabilities of class membership by number of deprivations (Row %)

Deprivation score	Class 1 (%)	Class 2 (%)	Class 3 (%)	Class 4 (%)
0	0	0	4	95
1	0	3	13	84
2	0	14	29	57
3	1	40	31	28
4	6	61	22	11
5	17	67	12	4
6	24	70	5	1
7	17	80	2	0
8	6	93	1	0
9	1	98	0	0
10	0	100	0	0

One advantage of FMMs over other methods is the possibility of computing a model-based deprivation score for each person in the sample. This is a very useful feature because it enables researchers to know the additive properties of the item—whether the sum of items A and C is equal to the sum of C and D.

For each person in the sample a severity score is computed according to their observed pattern. For example, it is possible to check what kind of sequences characterize people with three deprivations (Table 6) . For three deprivations there are 102 patterns or combinations in this sample. Although such table could be provided in this article, the aim is just to illustrate the kind of patterns that can be obtained by including the five combinations with the lower degrees of severity and the five with the highest. Table 6 (last column) show the estimated value of the factor for each combination, where the lower the value the lower the severity of deprivation. The results suggest that those lacking Minimum Social Protection Floor (MSPF), Food and Water suffer from less severe deprivation in comparison with those living in poor housing conditions (walls, flooring and roofing). This is a sensible result given that the IRT-model showed that those items characterize the severest forms of deprivation.

Table 6 is therefore useful to check the additivity properties of the deprivation items. For example, those lacking walls, flooring and roofing, even though they have the same deprivation score, are more severely deprived than those lacking food, MSPF and water. This is useful also in terms of policy recommendations. First, if one of the goals is to eradicate the severest forms of deprivation, it is possible to easily identify the patterns associated with the higher factor scores. Second, by checking which items typically characterize households or people with the severest forms of deprivation, it is possible to detect sets of deprivations that can be tackled jointly by specific policies (e.g. housing materials or public services).

**Table 6:** Selected patterns of multiple deprivation. People with a deprivation score = 3

Food	Education	MSPF	Ocercrowding	Walls	Flooring	Roofing	Fuel	Water	Sanitation	Factor
Top 5 lowest factor scores										
1	0	1	0	0	0	0	0	1	0	−0.168
0	0	1	1	0	0	0	0	1	0	−0.263
0	0	1	0	0	0	0	0	1	1	−0.282
0	0	1	1	0	0	0	0	0	1	−0.134
0	0	1	0	0	1	0	0	1	0	−0.106
Top 5 highest factor scores										
0	0	0	0	1	0	1	0	1	0	0.519
0	1	0	0	1	0	1	0	0	0	0.525
0	0	0	1	1	0	1	0	0	0	0.422
0	0	0	0	1	0	1	0	0	1	0.534
0	0	0	0	1	1	1	0	0	0	0.538

1 = Deprived, 0 = Not deprived

The results of a Mixture Model can be also utilized to analyse the relationship between income, multiple deprivation and severity. As previously mentioned, although it would be possible to fit a higher-order mixed-model, it would assess a rather different question—whether the intersection method identifies the poor. Table 7 shows the mean income per capita for each of the four classes as means to explore further the relationship between low/high income and severity of material deprivation. It is important to note that it would be possible to produce a table similar to Table 6, in order to test the additivity properties of the items and the relationship between income and different multiple deprivation patterns. Table 7 suggests, as expected, that people living in the worst forms of material deprivation tend to have low incomes (Class 1) and that people with low likelihood of experiencing multiple deprivation (Class 4) have a higher income. In fact, people in Class 4 are likely to have at least twice the income of people in Class 3 (4,273 pesos v 1,975 pesos).

**Table 7:** Mean income per capita by latent class (Mexican pesos, 2012)

Class	Mean	95 % CI	
1	1261	1240	1283
2	1627	1613	1640
3	1975	1948	2002
4	4273	4236	4309

In analysing the connection between income and material deprivation is vital to consider the possible casual links. In Mexico access to public services, education and minimum social protection floor have a weak connection with income. Patterns of multiple deprivation showing lacking these items, are likely to reflect the regressive nature of the Mexican welfare provision. In the case of adequacy of housing, the link is akin to Townsend’s theory in which people is deprived because they cannot afford certain items.

## Discussion and Conclusion

Poverty research is increasingly adopting the latent variable approach to analyse and understand poverty and deprivation (Szeles and Fusco 2013; Pirani 2013; Machado et al. 2009; Krishnakumar and Nagar 2008; Whelan and Maitre 2006, 2005). The purpose of this article is to contribute to the literature on the analysis of latent sub-groups experiencing different types of multiple deprivation. This area in poverty research has important implications for policy making. In identifying meaningful patterns of multiple deprivation is possible to inform what are the key areas for policies aiming at reducing, for example, the extremes forms of deprivation.

Unlike previous studies, a distinctive feature of this exercise is that it relaxes the assumption of conditional independence by using a Factor Mixture Model (Lazardfeld and Henry 1968). Such hybrid model has been recently proposed in the field of psychometrics to overcome some limitations of Latent Class Analysis and common factor analysis (Muthén 2007; Lubke and Muthén 2005; Muthén and Shedden 1999).

The main concern of this article was to apply Factor Mixture Modelling to the analysis of material deprivation using real data from Mexico 2012 in order to show its advantages and disadvantages. The article show that FMM’s have attractive properties that can be utilized for the analysis of multiple deprivation and severity.

The analysis shows that relaxing the assumption of local independence results in a better model. Therefore, the Factor Mixture Model seems to outperform the latent class model. There are two main differences between both models in terms of the conclusions that one can draw from both models. First, the number of classes is rather different, whereas the LCA model suggests the existence of six sub-groups, the FMM suggest four. Second, the FMM indicates that local independence does not seem to be an adequate assumption as the results suggest that severity fluctuates within and between class. This indicates that material deprivations are correlated within classes which is consistent with Townsend’s theory (Townsend 1987). Third, the FMM indicates that deprivation is not a discrete phenomenon, i.e. is a continuous one comprising different degrees of severity.

Factor mixture models have further potential applications for policy-making and the further analysis on severity of material deprivation. In particular, FMM’s help to explore the relationship between a deprivation score and different patterns of multiple deprivation. This feature could be useful to detect acute forms of material deprivation. From the policy perspective FMM’s are useful to identify patterns of multiple deprivation that can be subject to specific programmes. Such programmes can be developed to eradicate the severest forms of deprivation by looking at what are the sets of deprivations that tend to affect the extremely deprived.

One of the most attractive features of FMM’s is the possibility of analysing how severity of deprivations varies for different combinations of items. This has important application in terms of model-based deprivation scores, an issue that has been seldom investigated in the literature on severity. It also has potential application for the analysis of additivity of items as it shows how diverse combinations result in different degrees of severity of deprivation.

One vital question regarding such results is the following: How certain a researcher may be about selecting the correct model? Simulation studies suggest that when using continuous indicators almost always the available statistics lead to choose the correct model (Lubke and Neale 2006). The same nonetheless is not in all FMMs. In particular, when the thresholds are free to vary across classes the BIC criterion is very unreliable. The AIC seems to be much more trustworthy but still, according to simulations, there is a margin of error (Lubke and Neale 2008).

Factor mixture models are so flexible that they come at the expense of misspecification of the within-class factor structure. Because there is no absolutely reliable criterion for model selection, in exploratory settings it seems sensible to fix thresholds and loadings. In a confirmatory analysis on the number of latent sub-groups it is recommended to take precautions in the interpretation of the scope and implications of the results.
